# Screening Strategies for a Sustainable Endpoint for Gambiense Sleeping Sickness

**DOI:** 10.1093/infdis/jiz588

**Published:** 2019-12-26

**Authors:** M Soledad Castaño, Maryam Aliee, Erick Mwamba Miaka, Matt J Keeling, Nakul Chitnis, Kat S Rock

**Affiliations:** 1 Department of Epidemiology and Public Health, Swiss Tropical and Public Health Institute, Basel, Switzerland; 2 University of Basel, Basel, Switzerland; 3 Mathematics Institute, University of Warwick, Coventry, United Kingdom; 4 Zeeman Institute for Systems Biology and Infectious Disease Epidemiology Research (SBIDER), University of Warwick, Coventry, United Kingdom; 5 Programme National de Lutte contre la Trypanosomiase Humaine Africaine, Kinshasa, the Democratic Republic of the Congo; 6 School of Life Science, University of Warwick, Coventry, United Kingdom

**Keywords:** Democratic Republic of the Congo, elimination of transmission, gambiense human African trypanosomiasis, mathematical modeling, sleeping sickness

## Abstract

**Background:**

Gambiense human African trypanosomiasis ([gHAT] sleeping sickness) is a vector-borne disease that is typically fatal without treatment. Intensified, mainly medical-based, interventions in endemic areas have reduced the occurrence of gHAT to historically low levels. However, persistent regions, primarily in the Democratic Republic of Congo (DRC), remain a challenge to achieving the World Health Organization’s goal of global elimination of transmission (EOT).

**Methods:**

We used stochastic models of gHAT transmission fitted to DRC case data and explored patterns of regional reporting and extinction. The time to EOT at a health zone scale (~100 000 people) and how an absence of reported cases informs about EOT was quantified.

**Results:**

Regional epidemiology and level of active screening (AS) both influenced the predicted time to EOT. Different AS cessation criteria had similar expected infection dynamics, and recrudescence of infection was unlikely. However, whether EOT has been achieved when AS ends is critically dependent on the stopping criteria. Two or three consecutive years of no detected cases provided greater confidence of EOT compared with a single year (~66%–75% and ~82%–84% probability of EOT, respectively, compared with 31%–51%).

**Conclusions:**

Multiple years of AS without case detections is a valuable measure to assess the likelihood that the EOT target has been met locally.

Human African trypanosomiasis (HAT) is a neglected tropical disease affecting people in sub-Saharan Africa. Almost all human infections belong to the gambiense form, caused by the parasite *Trypanosoma brucei gambiense*, which is transmitted by tsetse vectors. Regular control activities in most endemic regions and reinforced surveillance have resulted in a dramatic and sustained decrease of new cases of gambiense HAT (gHAT), with global values falling from over 25 000 in 2000 to less than 1000 reported cases in 2018 [1].

Regular monitoring in recent years suggested that the goals set by the World Health Organization (WHO) of eliminating gHAT as a public health problem (Evidence into Public Health Policy [EPHP]) by 2020 could be achieved. The primary indicators for this target are (1) less than 2000 annual reported cases by 2020 and (2) a 90% reduction in the area reporting more than 1 case/10 000 people/year (calculated over a 5-year period), compared with the 2000–2004 baseline [2, 3]. An even more ambitious target to achieve elimination of transmission (EOT) across the continent is set for 2030 [3].

In the present study, we focus on the Democratic Republic of Congo (DRC) as the key country on which elimination of gHAT rests. The DRC has consistently contributed to the majority of the global reported case burden—in 2018, 660 of 953 total gHAT cases (69%) came from DRC [1]. Understanding when and how EOT might be achieved in DRC, and the link between underlying transmission and reported cases in the peri-elimination era, will be important for the planning and cessation of future control activities.

Across endemic gHAT regions, the mainstay of intervention strategies has been combined diagnosis and treatment of infection. Diagnosis occurs through a mix of passive surveillance (PS), with individuals self-presenting to fixed health facilities with HAT diagnostics, and active screening (AS), with mobile screening teams traveling to at-risk villages to test any person willing to participate. The intensity of AS has fluctuated over time; however, in 2016, 2.36 million people were tested serologically for gHAT during AS campaigns across Africa, and 56% of the reported cases came from AS [3]. The WHO’s recommendation is that endemic villages (with cases reported in the last 3 years) should be included for AS the following year. Those villages that stop AS should restart AS after case detections from the passive system or village members being identified as cases in AS that occurred elsewhere [4]; we refer to such screening as reactive screening (RS).

Cessation of routine AS is a challenging issue: although guidance exists at the village level [4], it is not easy to extrapolate this onto larger geographical units, particularly to the scale on which interventions are often planned. Furthermore, given the relatively high operational costs of AS, as prevalence declines, it will be appropriate at some point to stop AS to rely on PS and RS as control key strategies to detect the last remaining cases.

In this context, it is not only essential to know whether elimination targets can be reached in different settings using current or expanded strategies, but it is also important to consider the impact of cessation of large-scale activities after reaching the chosen stopping criteria. In this study, we focus on the indicator of zero reported cases, as a proxy for the achievement of EOT. For this approach, gHAT modeling can provide useful predictions of both reported cases and whether true EOT has been achieved, allowing an assessment of different control strategies.

Mathematical modeling of gHAT has been used to explore the impact of a variety of strategies on the timeline to achieve EPHP and EOT [5–10]. Strategies considered have included the following: different levels of AS, improving coverage of high-risk people in AS, reducing time to detection through improving PS, and vector control. The aforementioned models all suffer from being deterministic formulations—capturing “average” dynamics—and are therefore unable to reproduce the transition between low and zero transmission. Instead, deterministic models have used a proxy for EOT, such as <1 new infection per 100 000 or per 1 000 000 [7, 9, 10]. At extremely low prevalences, a stochastic model formulation is far more suitable because it captures the chance events governing transmission dynamics; it also produces integer-valued outputs for the number of cases and new infections and therefore provides better forecasting for EOT timelines.

In a previous study, a stochastic model of HAT was used to explore village-level persistence of infection in a high-endemicity region of former Bandundu province, DRC [11], providing an analysis of the probability that EOT had been achieved locally given there were no cases reported in the village. However, it is not trivial to extrapolate these village-level results onto larger administrative units, such as the health area (~10 000 people) and health zone (~100 000), to determine the probability of EOT if the region reports no cases for 1 or more years. In addition, we are interested in how such probabilities change over time. In the present study, we extend 2, previously deterministic, models to a stochastic framework to explore (1) when we expect to achieve EOT in at a health zone level in the high-endemicity former Bandundu province, DRC and (2) how the future intervention strategy could impact attainment and measurement of EOT.

## METHODS

The 2 models (Model S and Model W) used here were previously developed, independently, in a deterministic framework [5, 10, 12]. Both models share some structural similarities, eg, both are mechanistic models—explicitly modeling different stages of human infection and transmission occurring between humans and tsetse. Likewise, transmission dynamics within the models are influenced by individuals at high- and low-risk of exposure to tsetse bites, with high-risk people less likely to participate in AS activities.

Each of these deterministic models was first fitted to data from Bandundu. Model S was fitted to former Bandundu province-level screening patterns and case reporting from both AS and PS for the years 2000–2012; Model W was fitted to similar data from the Mosango health zone (within Bandundu province) for the period 2000–2016. Next, a stochastic implementation of these models (where each rate in the deterministic equations is translated into a stochastic process) was used to explore different scenarios at the health zone level: a “generic” health zone of former Bandundu province of population size 100 000 (Model S); and the specific health zone of Mosango (in former Bandundu province) with a population size of ~126 000 (in 2015) (Model W).

Further information of both models is provided in the Supplementary Information. In each scenario, the calibrated model is simulated forward to beyond 2040 to make projections of EOT under 7 medical-only strategies comprising PS, AS, and RS, with levels as indicated in Table 1. A further 7 strategies with higher coverage of AS are presented in the Supplementary Information. In all cases, a 3% annual growth rate was used for projections of human population. Although Model W made stochastic simulations throughout, Model S generated deterministic outputs for 2000–2012 and stochastic simulations afterwards. Models S and W used 1000 and 200 posterior parameter sets, respectively, with 100 and 1000 stochastic realizations performed for each. Calculations for the Model S were performed at the sciCORE (http://scicore.unibas.ch/) scientific computing core facility at the University of Basel.

**Table 1.** Overview of Future Medical-Only Strategies Considered

Strategy Name	AS Coverage^a^	Cessation of AS (2020 Onwards)	Restarting AS (ie, RS)	PS Coverage^b^	Cessation of PS
Nonstop	Mean	Never	N/A	Constant	Never
Stop 1	Mean	Stop after 1 year of zero reported cases	Never	Constant	Never
Stop 1_RS	Mean	Stop after 1 year of zero reported cases	After any passive detection	Constant	Never
Stop 2	Mean	Stop after 2 consecutive years of zero reported cases	Never	Constant	Never
Stop 2_RS	Mean	Stop after 2 consecutive years of zero reported cases	After any passive detection	Constant	Never
Stop 3	Mean	Stop after 3 consecutive years of zero reported cases	Never	Constant	Never
Stop 3_RS	Mean	Stop after 3 consecutive years of zero reported cases	After any passive detection	Constant	Never

Abbreviations: AS, active screening; NA, not applicable; PD, passive detection; PS, passive surveillance, RS, reactive screening.

^a^Mean of last 5 years of data.

^b^Same as last year of data.

Due to the timescale of gHAT infection, it is possible that some individuals could remain infectious without creating secondary cases for several years; to account for this, the year achieving EOT was defined as the first of 5 consecutive years of no new transmissions.

Models’ output consisted of the number of annual reported cases distinguished by detection strategy (ie, AS and PS) and new infections for 2020–2054, from which the probability of EOT was calculated. The positive predictive value (PPV) of EOT given that zero cases were reported in the year is also computed, providing a link between observable reporting and underlying transmission. To ensure a good estimation of the PPV, we excluded any years in which insufficient simulations have zero reported cases.

## RESULTS

**Figure 1.**
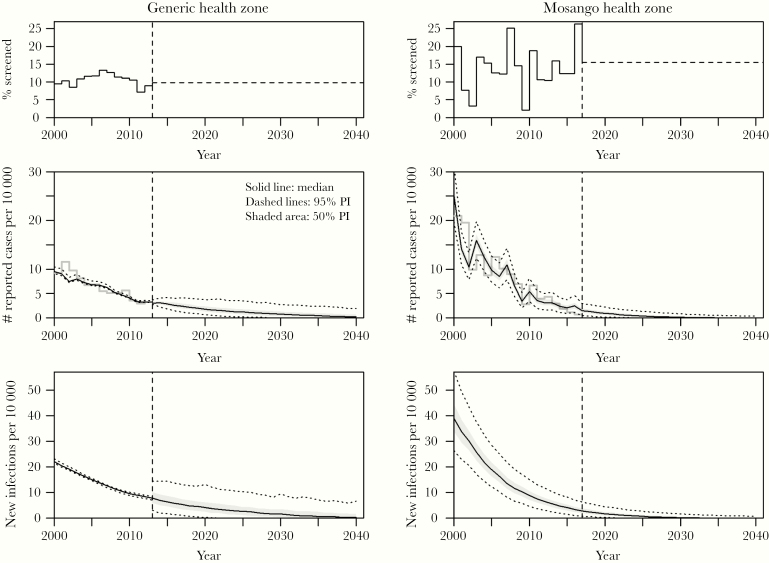
Time series dynamics in 2 health zones. Screening levels used by each model, including both data (continuous line) and projections (dashed line), are shown in the top row. Model outputs for up until 2040 are shown in the middle and bottom row only for strategy Stop 1 and include the following: estimations of the annual number of reported cases (middle) and underlying annual incidence (bottom). Each of these outputs are presented for a “generic” health zone of Bandundu province of 100 000 people (Model S, left side) and for Mosango health zone (~126 000 people, Model W, right side). Continuous black and dashed lines denote the model median fit and denote 95% credible intervals (CIs), respectively, whereas gray shading indicates 50% CIs. Vertical line indicates switch to projections. Same results for all the 7 strategies are shown in Supplementary Figure SI-1.

Higher variation is observed in AS data in the Mosango health zone than in the averaged generic health zone (GHZ) from Bandundu province (Figure 1, top panel), presumably representing the larger scale of Bandundu compared with Mosango. Screening levels used for Mosango forward projections, based in the last 5 years of data, are approximately 15.5% per year. Data at the province level used for the GHZ covers fewer years and is less variable, with an overall lower screening coverage and screening levels used for forward projections of 9.8% per year.

**Figure 2.**
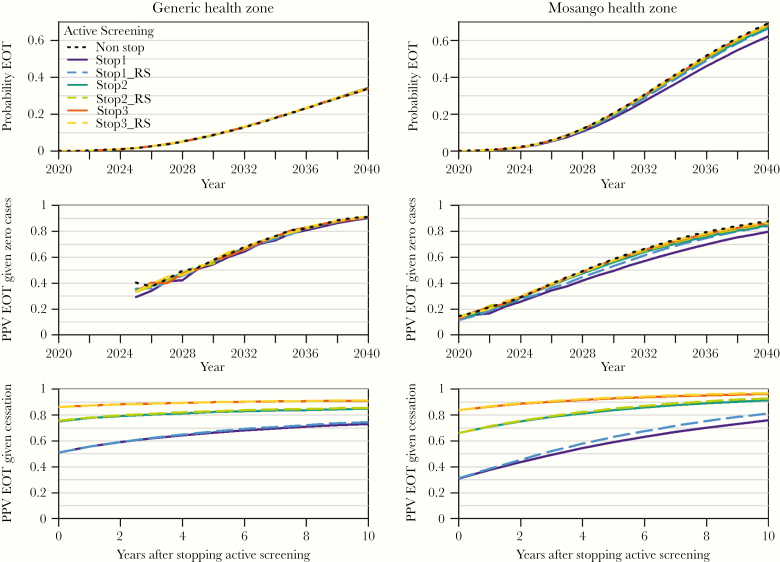
Probability of elimination of transmission (EOT) and positive predictive value (PPV) of zero case reporting. This figure shows the probability of EOT under each strategy for the period 2020–2040 (top panel), the PPV of zero reported cases to inform EOT (middle panel), and the PPV of years after the first stop in the 3 strategies that include this possibility (bottom panel). The left plots are for the generic health zone (GHZ), and the right plots are for Mosango health zone. For the GHZ, insufficient simulations (less than 300 of 100 000 runs) reached the condition of zero reported cases before 2025 and were excluded from the PPV analysis.

Figure 2 (middle and bottom panels) shows predictions of reported cases and transmission for Stop3_RS. Mosango started with more than two times the number of reported cases per 10 000 people in 2000 compared with the GHZ, with the underlying new infections estimated for this location doubling those estimated for the GHZ in that same year. There is a less steep decline in reported cases in the GHZ than in Mosango over the data period informing models, which is attributable to the lower level of AS. As a result, the transmission (new infections) falls to zero faster for the Mosango health zone. Predicted annual reported cases and new infections across all tested strategies are extremely similar to each other and are shown in Supplementary Figure S1.

For the GHZ, results suggest the that probability of achieving EOT by 2030 is approximately 10% (Figure 2, top panel). In contrast, for Mosango, EOT is likely to be achieved earlier, with the EOT probability of approximately 20% by 2030. This predicted difference between the 2 health zones is a result of higher assumed coverage of AS in projections for Mosango than for the GHZ, leading to a steeper decline in estimated transmission and therefore a greater chance of breaking transmission chains for Mosango. Results across all tested strategies are also similar to each other for Mosango; however, as expected, the no stopping strategy is marginally more likely to result in EOT than other strategies. Further simulations show that if the maximum percentage coverage (informed by the data) was used, the probability of EOT by 2030 for Mosango has a much more optimistic 29.9%–34.2% (Supplementary Figure SI-2), whereas for the GHZ of Bandundu province these values range 12.9%–13.3% (Supplementary Figure SI-3).

In Mosango, if zero cases are detected in 2020, then there is ~13% probability that EOT has been achieved; however, if zero detections occur later, there is a higher probability of approximately 55% (49.5%–58.6%) in 2030 and approximately 84% (79.8%–87.7%) in 2040 (Figure 2, middle panel). For the GHZ, a higher underlying transmission projected for the future leads to few simulations (less than 300 of 100 000 runs) with zero reported cases for 2020–2024, thus these years were not included in the analysis. The probability of EOT given zero cases for the GHZ is similar between strategies all along years and ranges 54.4%–57.9% by 2030, reaching values of approximately 90% (90.2%–91.8%) only by 2040.

Stopping AS after 3 consecutive years of zero reported cases produced a consistent higher probability of EOT for any number of years after AS cessation compared with the other strategies in both health zones. These probabilities increase very slowly with increasing time, starting at 86.1% for the GHZ and 83.7% for Mosango (Figure 2, bottom panel). Because more years with zero reported cases were required to stop AS, the improvement in the probability of EOT associated to adding RS for each of these was weaker, with Stop3 and Stop3_RS producing similar values.

Probabilities of EOT for either Stop2 or Stop2_RS strategies reached 80% after 2 and 4 years of stopping AS in the GHZ and Mosango health zone, respectively. Although for the GHZ further increase with time was slow (~75.5% for 10 years after AS cessation), for Mosango this increase was faster and attained values >90% between 9 (Stop2_RS) and 10 (Stop 2) years after AS cessation. As expected, probabilities of EOT for Stop 1 strategy were the lowest, and these reached values below 80% even 10 years after stopping AS in both health zones.

## DISCUSSION

Projections from both models suggest that, surprisingly, the health zone level cessation of AS activities had little impact on the overall transmission dynamics. This can be explained by the lag in infection in reporting, with the number of reported cases typically reaching zero several years after the last transmission event. By the time zero cases are reported in the health zone, so few infections remain that eventual stochastic extinction is likely without further AS.

Despite the expected decreasing trend in the number of reported cases and underlying incidence under current levels of medical-based strategies, for all criteria for cessation of AS in both the generic and Mosango health zone, there is not high confidence that this trend would be enough to achieve the 2030 target of interrupting transmission. Because the GHZ results from aggregated data at a province level, we could expect even more pessimistic scenarios for some health zones in Bandundu. Mosango seems able to reach EOT by 2040 with >70% probability if maximum AS is continued alongside PS for most stopping strategies, but, although this level of screening has a moderate probability (~35%) of meeting EOT in the desired timeframe (by 2030), alternative strategies may be preferred to improve the chance of meeting the goal.

Because gHAT is known to be a remarkably persistent disease, even at extremely low prevalence, it was not previously clear whether zero case detection would be sufficient to lead to disease fade out if AS stopped. Despite the limited difference in outcomes between strategies, the strategy requiring 3 years of zero case reporting does provide much more valuable information to assess whether EOT has been met. This is the strategy for AS cessation recommended by the WHO at the village level [4], and methods here provide a quantitative scheme to compare this recommendation against less restrictive AS stopping criteria. We highlight that key to these predictions is the assumption of a continued and sustained PS system, and that a more complete analysis of EOT probabilities using zero reported cases should include the effects of different PS levels. Several unknown variables challenge this approach including underreporting levels and the likely reduction in the number of health facilities able to perform gHAT diagnosis as the cases reporting approaches zero.

The Bandundu province data used for the GHZ averages out variation in endemicity and activities across health zones, with subsequent effects on estimates that would not correspond with specific health zones, as in the Mosango example. Furthermore, there is not only a potential need to adapt proxies for EOT to different endemicity regions, but also to the appropriate spatial scale. For instance, when considering dynamics at the village scale (in the same region of DRC—Yasa-Bonga and Mosango health zones, Bandundu province), typically 3 or more years of zero case detection are required to have >90% confidence of EOT for typical AS coverage in settlements of approximately 2000 people [11]. The coverage of AS appears to not only speed up the time to EOT, but also to slightly improve the PPV of using zero case detection to measure EOT at the health zone level (see Supplementary Figures S2 and S3) as well as at the village scale [11].

Estimates from both health zone settings suggest that achieving the WHO goal of eliminating gHAT transmission by 2030 would require a step change in the level of surveillance and the use of additional interventions in persistent regions of DRC. Currently available interventions that could help accelerate progress towards EOT include the following: better targeting of high-risk populations by door-to-door AS (used in Côte d’Ivoire [13] and some regions of DRC); minimobile teams reaching areas otherwise not accessible; greater accessibility to health facilities and gHAT-testing; and suppression of the local tsetse population (with the “tiny target” method yielding >80% reduction in vector density in some foci in Guinea [14], Chad [8], and Uganda [15]). Simulation modeling work suggests that targeted AS and/or vector control could dramatically accelerate reduction in transmission, particularly in persistent hotspots [6, 7, 9]. Fexinidazole, an all-oral 10-day treatment for both stages of HAT (excluding severe cases), has recently been approved [16] and could improve the ease of access to treatment, thus contributing to reaching EOT deadlines.

The model variants used in this study do not account for (1) possible animal reservoirs or (2) “latent” infections in humans that do not lead to disease. Although it remains unclear whether either of these types of infection routinely contribute to transmission, it is a concern that their presence could hinder efforts to achieve EOT [17], in the same manner that dog reservoirs may threaten Guinea worm eradication [18]. Modeling of animal reservoirs to date indicates that rather than preventing EOT, it could impact the timescale in which it can be achieved [5, 12], and there might be a higher chance of recrudescence of disease if screening is stopped early [19]. Future stochastic models of “cryptic” HAT reservoirs would be best able to assess their impact on time to EOT and using zero case reporting as a proxy.

## CONCLUSIONS

We have generated stochastic projections of gHAT dynamics in DRC at the health zone level using 2 different models and data sets to explore the link between different AS cessation criteria based on zero reported cases and the probability of reaching EOT. We found that AS cessation based on different zero case reporting related criteria had a limited impact on the time evolution of underlying transmission, and that 3 years of zero cases provides valuable information to assess the probability that transmission has been interrupted.

## Supplementary Data

Supplementary materials are available at *The Journal of Infectious Diseases* online. Consisting of data provided by the authors to benefit the reader, the posted materials are not copyedited and are the sole responsibility of the authors, so questions or comments should be addressed to the corresponding author.

jiz588_suppl_Supplementary-Figure-S1

jiz588_suppl_Supplementary-Figure-S2

jiz588_suppl_Supplementary-Figure-S3

jiz588_suppl_Supplementary_Information

## Notes

***Acknowledgments.*** We thank the World Health Organization human African trypanosomiasis (HAT) team for facilitating access to the HAT Atlas data [3] and for helpful discussion and comments on this manuscript.

***Disclaimer.*** The views, opinions, assumptions or any other information set out in this article are solely those of the authors and should not be attributed to the funders or any person connected with the funders. The funders had no role in study design, data collection and analysis, decision to publish, or preparation of the manuscript.

***Supplement sponsorship.*** The authors acknowledge funding of the NTD Modelling Consortium by the Bill and Melinda Gates Foundation (OPP1184344).

***Potential conflicts of interest.*** All authors: No reported conflicts of interest. All authors have submitted the ICMJE Form for Disclosure of Potential Conflicts of Interest.

***Financial support.*** This study was funded by the Bill & Melinda Gates Foundation (BMGF) (https://www.gatesfoundation.org/) in partnership with the Task Force for Global Health (https://www.taskforce.org/) through the Neglected Tropical Diseases (NTD) Modelling Consortium (OPP1184344; to M. S. C., M. A., M. J. K., N. C., and K. S. R.). K. S. R. and M. J. K. also were funded by BMGF through the HAT Modelling and Economic Predictions for Policy project (OPP1177824).

Presented in part: 11th European Congress on Tropical Medicine and International Health, Liverpool, UK.
