# Supplementary material for: Screening Strategies for a Sustainable Endpoint for Gambiense Sleeping Sickness
Source: J Infect Dis. 2019 Dec 26;221(Suppl 5):S539–45. doi: 10.1093/infdis/jiz588 (PMC7289553; doi:10.1093/infdis/jiz588)
Supplement: jiz588_suppl_Supplementary-Figure-S1 [file jiz588_suppl_supplementary-figure-s1.pdf]

Screening strategies for a sustainable endpoint for gambiense sleeping sickness

Supplementary Information 1

Model output for the different strategies considered

In Figure 1 of the main document we show model outputs only for the strategy based on stopping active screening after one year of zero reported cases (Stop1). The figure below shows model outputs for all strategies considered. Given the extremely similar estimates for the number of reported cases and annual new transmission across strategies, most of lines and shaded areas overlap.

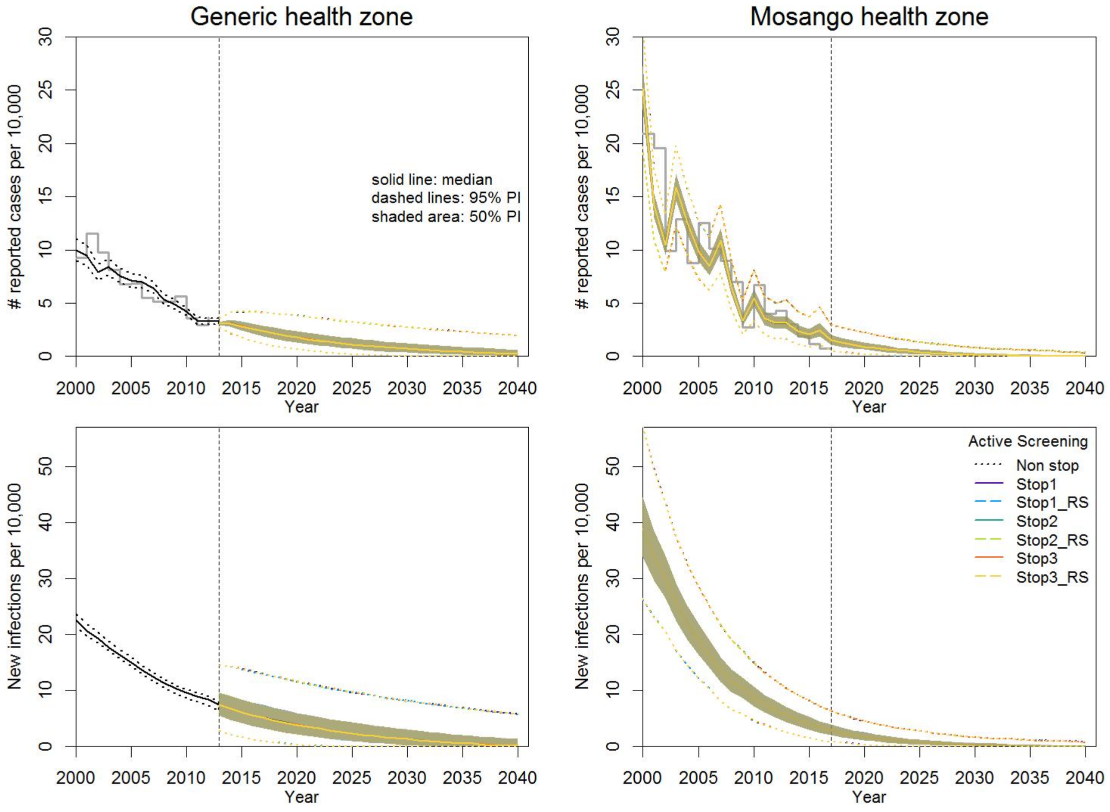

Figure 1: **Time series dynamics in two health zones.** Model outputs for up until 2040 are shown for all strategies considered and include: estimations of the annual number of reported cases (top) and underlying annual incidence (bottom). Each of these outputs are presented for a "generic" health zone of Bandundu province of 100,000 people (Model S, left side), and for Mosango health zone (~126,000 people, Model W, right side). Continuous black and dashed lines denote the model median fit and denote 95% credible intervals (CIs) respectively, while grey shading indicates 50% CIs. Vertical line indicates switch to projections.
