# Supplementary material for: Screening Strategies for a Sustainable Endpoint for Gambiense Sleeping Sickness
Source: J Infect Dis. 2019 Dec 26;221(Suppl 5):S539–45. doi: 10.1093/infdis/jiz588 (PMC7289553; doi:10.1093/infdis/jiz588)
Supplement: jiz588_suppl_Supplementary-Figure-S3 [file jiz588_suppl_supplementary-figure-s3.pdf]

Screening strategies for a sustainable endpoint for gambiense sleeping sickness

Supplementary Information 3

Models output for two different assumed levels of active screening ("generic" health zone)

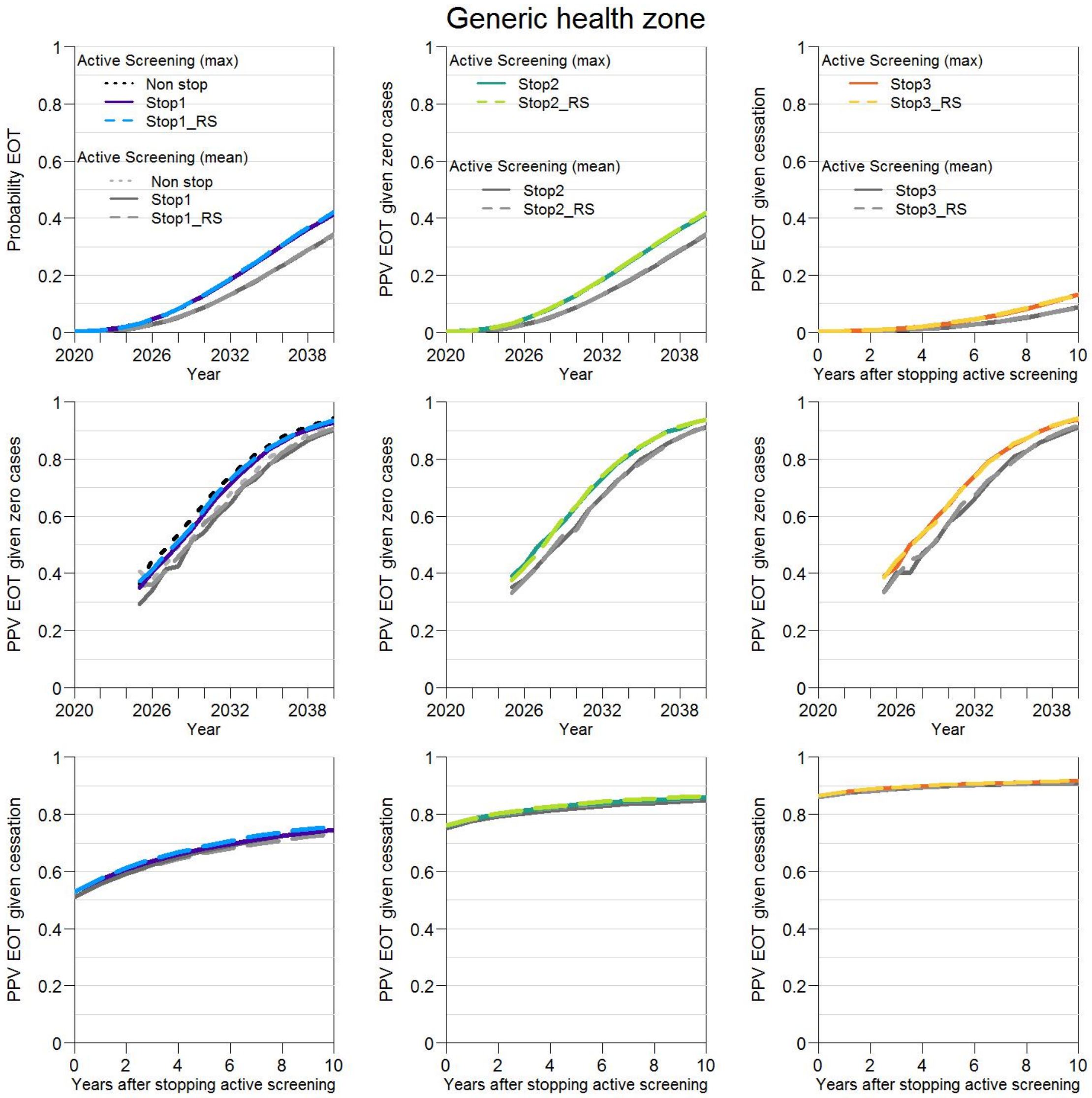

Figure 1: Comparison of mean (gray lines) and maximum (coloured lines) percentage coverage active screening in elimination prediction between different strategies for the Bandundu generic health zone (Model S).
