# Supplementary material for: Screening Strategies for a Sustainable Endpoint for Gambiense Sleeping Sickness
Source: J Infect Dis. 2019 Dec 26;221(Suppl 5):S539–45. doi: 10.1093/infdis/jiz588 (PMC7289553; doi:10.1093/infdis/jiz588)
Supplement: jiz588_suppl_Supplementary_Information [file jiz588_suppl_supplementary_information.pdf]

Supplementary information: Models used in this analysis

Screening strategies for a sustainable endpoint for gambiense sleeping sickness

M. Soledad Castaño[1,2,*],Maryam Aliee[3,4,*], Erick Mwamba Miaka[5]
Matt J. Keeling[3,4,6], Nakul Chitnis[1,2], Kat S. Rock[3,4]
* Authors contributed equally
1 Department of Epidemiology and Public Health,
Swiss Tropical and Public Health Institute, Basel, Switzerland
2 University of Basel, Basel, Switzerland
3 Mathematics Institute, University of Warwick, Coventry, CV4 7AL, UK
4 Zeeman Institute for Systems Biology and Infectious Disease Epidemiology Research,
University of Warwick, Coventry, CV4 7AL, UK
5 Programme National de Lutte contre la Trypanosomiase Humaine Africaine,
Kinshasa, the Democratic Republic of the Congo
6 School of Life Science, University of Warwick, Coventry, CV4 7AL, UK

1 Model S

1.1 Description

1.1.1 Deterministic model

The deterministic Model S used here was presented and described in (2) and is a variant of the HAT transmission model originally published in (19). The model consists of a system of coupled ordinary differential equations (ODEs), with compartments for tsetse, animal and human populations. These three different host types are modelled for two different settings corresponding to a low transmission area (e.g. the village, L) and a high transmission area (such as river banks or plantations, H) that enable accounting for heterogeneity in exposure to tsetse bites. The population size for tsetse, animal or humans in each setting i ($i = \{L, H\}$) is assumed to be stable by allowing the associated birth terms to compensate deaths in all the compartments. Tsetse and animal populations always stay within their setting (for example, tsetse in low transmission settings always remain in the low transmission setting and animals in high transmission settings always remain in the high transmission setting). Similarly, humans in low transmission settings always remain in low transmission setting. However, humans in the high transmission setting move back and forth between the high and low transmission settings spending a fixed amount of time in each one (to model, for example, the movement of high risk individuals between villages and plantations) — as shown in Figure 1.

Five compartments describe humans in any of the two settings: susceptible (S_{hi}); exposed or incubating (E_{hi}); infected with the first stage of the disease (I_{h1i}); infected with the second stage of the disease, where trypanosomes have reached the cerebro-spinal fluid (I_{h2i}); and treated (T_{hi}). The total human population in setting i is $N_{hi} = S_{hi} + E_{hi} + I_{h1i} + I_{h2i} + T_{hi}$.

Tsetse populations are divided into susceptible (S_{vi}); teneral (U_{vi}); exposed (E_{vi}); and infected (I_{vi}), so that the vector population is $N_{vi} = S_{vi} + U_{vi} + E_{vi} + I_{vi}$.

As in (2), in this model implementation: *i)* animals do not contribute to transmission, thus animal populations are modelled as constant parameters, N_{ai}, and only form a sink for tsetse bite; *ii)* both stages (rather than only stage 1) of the disease are exposed to tsetse fly bites; *iii)* an additional compartment in the vector dynamics, U_i, accounts for the teneral effect — a reduction of infectivity with time — such that on average tsetse are only infectious for the first five days after emergence. These changes were made with respect to the original version (19) to provide a more realistic representation of the transmission dynamics. A schematic of the model is shown in Figure 1.

1.1.2 Stochastic implementation

Epidemiological deterministic models including previous implementations of Model S (19; 15; 2) have the shortcoming that they do not capture rare events and do no account for the discrete nature of populations, and are therefore unable to reproduce the transition between extremely low prevalence and zero transmission. For such situations, a discrete stochastic model formulation is more suitable as it captures the stochastic nature of events involved in transmission dynamics while producing integer outputs (e.g. number of cases and new infections here) that enable a clearer definition of elimination of transmission (and subsequent forecasting of elimination timelines) than in deterministic ODE models where arbitrary thresholds must be defined.

In the stochastic formulation of the ODE model described in 1.1.1, we model all human host, animal host and tsetse fly populations as discrete numbers, and all individuals move probabilistically between compartments at varying intervals of time. Any process governing the HAT transmission dynamics is considered stochastic, with terms in the compartmental model being now considered as probabilities at which an event occurs.

We implemented the direct method of the stochastic simulation algorithm (SSA; also known as Gillespie method (10)). In the direct method of the SSA, all posible events in the HAT transmission dynamics have an associated rate given by the associated term in the deterministic ODEs. For example, if γ represents the rate at which humans infected in the stage 1 of the disease (I_1) move to the second stage of the disease (I_2), thus γI_1 represents the rate R for the event "progression to stage 2 of the disease".

In order to simulate one stochastic realisation under the direct method, for i possible events with associated rate R_i, at any time t:
(a) we determine the time $t+\tau$ at which the next event happens, with τ an exponentially distributed random number scaled by the sum of all process rates, $\Sigma_i R_i$; and
(b) we decide which that event will be: the event that happens next is obtained through drawing a process randomly from all possible processes according to their respective probabilities $\frac{1}{R_i}$.

In the present analysis, 1000 posterior parameter sets (see 1.4) were used along with the fixed parameters to obtain 100,000 realisations of the stochastic model (100 realisations for each parameter set).

Simulations up to 2012 (end of data used for calibration) were deterministic, i.e. by numerical solving the ODE system, with further projections using the stochastic implementation described in this section.

Deterministic model output at 2012 was scaled up to a 100,000 human population (to represent the approximate size of health zones in Bandundu) and round to integer values before running stochastic simulations of the forward discrete model.

The discrete stochastic model was then run until 2054 in order to allow evaluation of the positive predictive value of zero case detection to predict elimination of transmission (EOT) (Figure 2 in

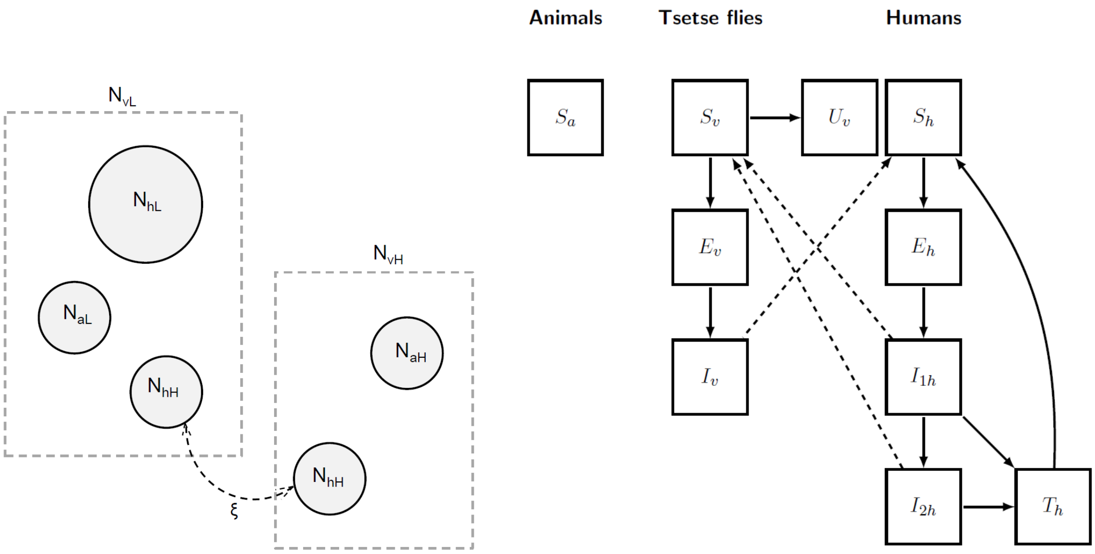

Figure 1: **Schematic of Model S**. Left: model population structure. Human populations are composed by a stationary population (N_{hL}) that remains in low exposure habitats (e.g., a village), and a smaller population (N_{hH}) which commute and spend a proportion ξ of their time in a potentially high exposure setting (e.g., a plantation). Each habitat also contains tsetse (N_{vL} and N_{vH}) and non-human vertebrate animal populations (N_{aL} and N_{aH}). Right: schematic of infection dynamics, subscripts $i = \{L, H\}$ were removed for easy reading. Compartmental diagram highlights the transmissions between states of infection of the tsetse and human populations, with solid lines indicating transition between compartments, and dashed lines representing transmission rates. Animals cannot transmit infection thus acting as a sink for tsetse bite. Note that in the low-risk transmission setting, both human populations are exposed to tsetse bites. Figure adapted from (19).

the main text), with EOT defined as five consecutive years of no new transmission. The code and posterior parameters used are attached.

1.2 Screeening

Test and treat interventions encompass both active screening and passive surveillance. Passive detection is represented by a continuous stage-specific detection rate and removes infected people from both low- and high-risk settings whilst active screening only recruits people in the low risk setting. With Bandundu province staged data suggesting an enhanced passive surveillance system, an improvement with time was included in the detection rate of stage 2, r_2 by multiplying the fitted constant of proportionality, c_2, by the proportion of people screened through passive surveillance as informed by data which showed an increasing trend.

Active screening was modelled as as a pulsed activity taking place in the first month of each year as this is a more realistic representation of actual campaigns than the continuous rate of active screening used previously in this model (19). We followed (1) to relate a proportion, d, of humans effectively screened in a given a year and the daily removal rate $r_{as}^{\text{continuous}}$ as $d = 1 - \exp(-365 r_{as}^{\text{continous}})$. Thus, for the pulsed active screening, we get: $r_{as}^{\text{pulsed}} = 12 r_{as}^{\text{continous}} = -(12/365)\ln(1-d)$. Screening levels were informed from data; estimates for the population of Bandundu were taken from (11) for the period corresponding to calibration, and a 3% annual growth was assumed for projections.

The unknown proportion of the population at risk of infection in Bandundu province is included via ϵ, such that $d(t) = \frac{X_s(t)}{\epsilon N_B(t}$, where $X_s(t)$ indicates number of people screened in year t, and $N_B(t)$ indicates Bandundu province population in year t. With no additional data enabling estimating ϵ, this parameter was set as a constant value.

1.3 Parameter values

Except ϵ and α which are parameters new to the model and that were assumed fixed since their incorporation in (2), model parameters assigned fixed were taken from Model S posteriors (median) in (15), and are described in Table 1

Table 1: **Model parameterisation (fixed parameters).** Notation, a brief description, and the used values of fixed parameters in Model S.

Notation	Description	Value
α	Rate at which tsetse become non-teneral (i.e. cannot get infectious)	73 year^{-1}
A/H_1	Density of animals relative to humans in area L	0.7
A/H_2	Density of animals relative to humans in area H	0.9
b	Proportion of infective bites leading to infection in humans and animals	0.8
c_{ai}	Proportion of bites on an infective animal of type i that lead to a mature infection in flies	0
δ	Rate at which treated humans return to the susceptible class	2.19 year^{-1}
δ_a	Rate of loss of immunity in animal hosts	1.095 year^{-1}
ϵ	Proportion of population at risk	0.7
η	Rate at which hosts move from the incubating stage	31.025 year^{-1}
f	Inverse of duration of feeding cycle; or biting rate	121.545 year^{-1}
γ	Rate of progression to stage 2 in humans	0.365 year^{-1}
γ_{aL}	Rate of progression to the immune class in animal hosts of type L	0.73 year^{-1}
γ_{aH}	Rate of progression to the immune class in animal hosts of type H	0.6935 year^{-1}
μ	Death rate of humans due to natural causes	0.02 year^{-1}
μ_{ai}	Death rate of animal host of type *i*	0.511 year^{-1}
μ_t	Death rate of humans due to treatment	0 year^{-1}
μ_v	Death rate of tsetse	10.95 year^{-1}
ν	Inverse of the extrinsic incubation period	13.505 year^{-1}
r_1	Removal rate of infected humans in stage 1 due to treatment (passive detection)	4.6144 year^{-1}
σ	Biting preference for humans	0.4
σ_{ai}	Biting preference for animal in the setting i	0.3
ξ	Proportion of time spent i the high risk region by commuters	0.62

1.4 Summary of previous fitting

The deterministic ODE version of Model S was calibrated to province level data for Bandundu (Democratic Republic of Congo) using an Aproximate Bayesian Computation (ABC) algorithm in a previous work (2) fitting six parameters. The data consisted of annual, staged reported cases for 2000-2012 from active screening and passive detection (indicated as fit to "staged data' in (2)). A summary of the fitted parameter posteriors is given in Table 2.

Table 2: **Model parameterisation (posterior parameters).** Notation, a brief description, the median values, and the 95% certainty intervals of fitting parameters in Model S.

Notation	Description	Value		Unit
		Median	95% CI	
c_h	Proportion of bites on an infective human that lead to a mature infection in flies	3.2134 $\times 10^{-3}$	[2.554, 3.9612] ($\times 10^{-3}$)	-
c_2	Constant of proportionality relating proportion of population screened to detection rate	22.3726	[15.2063, 33.0127]	[year^{-1}]
μ_γ	Disease-induced death rate or rate of leaving the recovered state for humans	0.6189	[0.4852, 0.7261]	[year^{-1}]
m_{HL}	Ratio of humans in the high exposure to low exposure environment	0.2468	[0.1508, 0.2951]	-
vh_L	Number of vectors per human in area L	3.1426	[2.4479, 3.9298]	-
vh_H	Number of vectors per human in area H	3.6195	[2.8221, 4.4467]	-

2 Model W

2.1 Description

Model W provides a stochastic version of the ODE model presented in (16; Crump et al.). The original model describes dynamics of gHAT transmission explicitly considering compartments of humans and tsetse. Figure 2 shows a schematic description of HAT dynamics in this model. Humans can be exposed and subsequently infectious by a bite of an infectious tsetse. They progress through different stages of the infection (stage 1 and stage 2) with different rates (σ_H and ϕ_H respectively). On the other side, tsetse vectors can become exposed and subsequently infectious if they bite an infectious human. Infected people may be detected by passive and active screening (more details in 2.2), followed by hospitalisation and recovery. Here, we consider a version of the model where humans are partioned into two compartments of (i) low-risk and participating in the active screening, and (ii) high-risk and non-participating in active screening. We assume there are no animal reservoirs although animals receive some proportion of tsetse bites. For simplicity, we assume the total population of humans to be constant, however, we take into account growth of population (3%) for comparison to the observed data (more details in 2.2).

In this stochastic model, individual humans are assigned to different compartments associated with infection/disease status and can transition between them. We describe system dynamics by random events captured by a tau-leap approximation. Table 3 explains different events and the corresponding

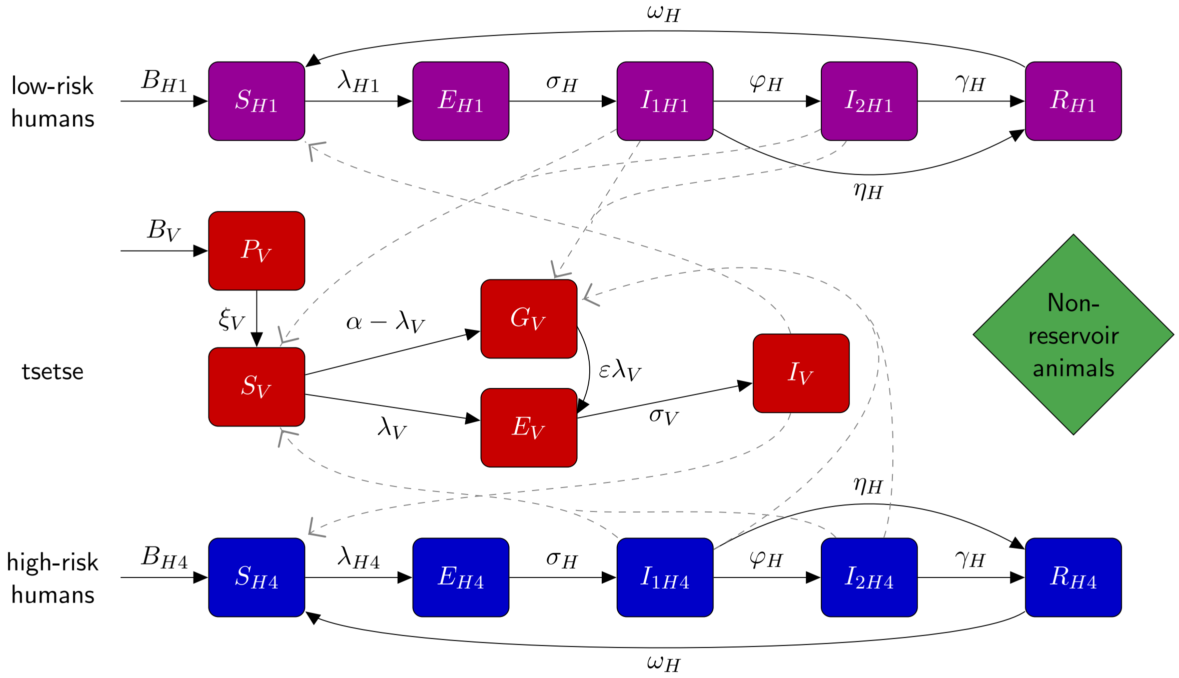

Figure 2: **Schematic of Model W to describe gHAT infection dynamics.** This multi-host model of HAT takes into account high- and low-risk groups of humans and their interactions with tsetse vectors. Each group consists of different compartments: Susceptible humans S_{Hi} can become exposed on a bite of an infectious tsetse. Exposed people E_{Hi} progress to become the stage 1 infected people and eventually stage 2 (if not detected in active screening), and once treated they recover by hospitalization R_{Hi}. Active screening can accelerate treatment rate of infected people. Here we assume high-risk group does not participate in active screening. By biting an infectious person, tsetse can become exposed and subsequently infectious, E_V and I_V. G_V represents the tsetse population not exposed to *Trypanosoma brucei gambiense* in the first blood-meal and are therefore less susceptible in the following meals. Rates are shown by Greek letters associated with arrows. Animal reservoir is not considered. This figure is taken from (Crump et al.) and adapted from the original model schematic (16).

Table 3: Model formulation (human dynamics)

**Event**	**Transition**	**Rate**
Recovery from hospitalisation	$s_{Hi} \to s_{Hi} + 1, r_{Hi} \to r_{Hi} - 1$	$\omega_H r_{Hi}$
Natural death of hospitalised	$s_{Hi} \to s_{Hi} + 1, r_{Hi} \to r_{Hi} - 1$	$\mu_H r_{Hi}$
Exposure of susceptibles	$s_{Hi} \to s_{Hi} - 1, e_{Hi} \to e_{Hi} + 1$	$f_{Hi} \alpha m_{eff} s_{Hi} I_V / N_{Hi}$
Progression to Stage 1 infection	$e_{Hi} \to e_{Hi} - 1, i_{1Hi} \to i_{1Hi} + 1$	$\sigma_H e_{Hi}$
Natural death of exposed	$e_{Hi} \to e_{Hi} - 1, s_{Hi} \to s_{Hi} + 1$	$\mu_H e_{Hi}$
Progression to Stage 2 infection	$i_{1Hi} \to i_{1Hi} - 1, i_{2Hi} \to i_{2Hi} + 1$	$\varphi_H i_{1Hi}$
Natural death of Stage 1 infection	$i_{1Hi} \to i_{1Hi} - 1, s_{Hi} \to s_{Hi} + 1$	$\mu_H i_{1Hi}$
Treatment or death from Stage 2 infection	$s_{Hi} \to s_{Hi} + 1, i_{2Hi} \to i_{1Hi} - 1$	$\gamma_H i_{2Hi}$
Natural death of Stage 2 infection	$i_{2Hi} \to i_{2Hi} - 1, s_{Hi} \to s_{Hi} + 1$	$\mu_H i_{2Hi}$

rates that lead to one person transitioning from one compartment to another one. Within this framework, the number of events happening in a time interval τ is chosen randomly from a Poisson distribution with the mean equal to the event rate multiplied by τ. We use time interval of a day in the tau-leaping algorithm that is shown to be a sensible choice for gHAT dynamics (8).

To avoid the need to disaggregate the parameter m_{eff} - which was used to non-dimensionalise the previous ODE system - into its component parts (p_H and N_V/N_H), we keep vector dynamics the same as the original model, described by a set of ODEs:

$$
\begin{aligned}
\frac{dS_V}{dt} &= \mu_V N_H - \alpha S_V - \mu_V S_V \\
\frac{dE_{1V}}{dt} &= \alpha p_V (f_{H1}\frac{I_{1H1}+I_{2H1}}{N_{H1}} + f_{H2}\frac{I_{1H2}+I_{2H2}}{N_{H2}})(S_V + \varepsilon G_V) - (3\sigma_V + \mu_V)E_{1V} \\
\frac{dE_{2V}}{dt} &= 3\sigma_V E_{1V} - (3\sigma_V + \mu_V)E_{2V} \\
\frac{dE_{3V}}{dt} &= 3\sigma_V E_{2V} - (3\sigma_V + \mu_V)E_{3V} \\
\frac{dI_V}{dt} &= 3\sigma_V E_{3V} - \mu_V I_V \\
\frac{dG_V}{dt} &= \alpha \left(1 - p_V \left(f_{H1}\frac{I_{1H1}+I_{2H1}}{N_{H1}} + f_{H2}\frac{I_{1H2}+I_{2H2}}{N_{H2}}\right)\right) S_V \\
&- \alpha p_v \left(f_{H1}\frac{I_{1H1}+I_{2H1}}{N_{H1}} + f_{H2}\frac{I_{1H2}+I_{2H2}}{N_{H2}}\right) \varepsilon G_V - \mu_V G_V. \qquad (2.1)
\end{aligned}
$$

This is a legitimate assumption due to the high population of vectors and their short life cycle compared to humans. We solve these equations with the help of Runge-Kutta methods.

2.2 Screening

This model accounts for the possibility of detecting of infected humans through passive and active screening. Passive screening describes potential visits of people to fixed medical centers for testing. In the stochastic version, we identify the number of people participating in passive screening by a tau-leaping approximation with rates proportional to η_H and γ_H corresponding to the first and second stages of the disease (Table 3).

Before 1998 (pre-active screening) it was assumed that passive detection was less effective than after activities began, and only so identified stage 2 individuals at a rate γ_{H0}, which is smaller than the stage 2 passive detection rate from 1998 onwards, γ_H.

Following previous modelling work using gHAT data from former Bandundu province (2; Crump et al.), there is a strong signal from epidemiological staging data that passive screening has improved during the time period from 2000–2016. To capture the steadily increasing trend in the proportion of stage 1 to stage 2 passive detections, the model utilises the following formula:

$$d_1(Y) = \eta_H \left[1 + \frac{d_{\text{amp}}}{1 + \exp\left(-d_{\text{steep}}(Y - d_{\text{change}})\right)}\right], \qquad (2.2)$$

where Y is the year and η_H is the stage 1 passive detection rate. Parameters dictating the amplitude, steepness and switching year can be found in Tables 4 and 5. d_{change} was established from fitting Model W to province-level data and is fixed. The other two parameters have been estimated through fitting to the health zone level data for Mosango.

In our current approach, active screening is considered to be a random procedure to detect infected humans within the people participating voluntarily. The number of people picked up from each compartment is given by a random number chosen from a binomial distribution. Similar to the previous models, we allow for the imperfect nature of the tests by considering sensitivity of tests to detect true cases and specificity to observe false positive cases. Specificity is set to one after 2015 due to improvement in confirmatory quality control (Crump et al.).

Using a similar approach to the previous ODE models, we consider the same level of screening as reported between 2000–2016. It is assumed, as in much of the previous published studies using this model, that active screening began in 1998 and achieved the same number of people screened as in 2000 (the first year of data). After 2016, we use the average and maximum percentage of screened people between 2012–2016 in different strategies.

2.3 Parameter values

As in previous versions of Model W (16; 17; 15; 8; Crump et al.; 2), some parameters with estimates available in the literature were assigned fixed values. Fixed values are given in Table 4. The other parameter values were taken from posterior distributions by fitting the model to data (see 2.4 for an outline of methods and summary of statistics of parameters).

Table 4: **Model parameterisation (fixed parameters).** Notation, a brief description, and the used values of fixed parameters in model W.

Notation	Description	Value	
N_H	Total human population size (in 2015)	121,433	(13)
B_H	Total human birth rate	$= \mu_H N_H$	
μ_H	Natural human mortality rate	5.4795×10^{-5} days^{-1}	(20)
σ_H	Human incubation rate	0.0833 days^{-1}	(18)
φ_H	Stage 1 to 2 progression rate	0.0019 days^{-1}	(4; 5)
ω_H	Recovery rate/waning-immunity rate	0.006 days^{-1}	(12)
Sens	Active screening diagnostic sensitivity	0.91	(3)
B_V	Tsetse birth rate	0.0505* days^{-1}	(17)
ξ_V	Pupal death rate	0.037 days $^{-1}$	
K	Pupal carrying capacity	$= 111.09N_H^{\dagger}$	(17)
$\mathbb{P}$(pupating)	Probability of pupating	0.75	
μ_V	Tsetse mortality rate	0.03 days^{-1}	(18)
σ_V	Tsetse incubation rate	0.034 days^{-1}	(9; 14)
α	Tsetse bite rate	0.333 days^{-1}	(21)
p_V	Probability of tsetse infection per single infective bite	0.065	(18)
ε	Reduced non-teneral susceptibility factor	0.05	(16)
f_H	Proportion of blood-meals on humans	0.09	(6)
d_{change}	Switching year for stage 1 passive improvement	2008	(Crump et al.)

2.4 Summary of previous fitting

The deterministic ODE version of Model W was fitted to health-zone-level data for Mosango using an adaptive Metropolis-Hastings MCMC algorithm (Crump et al.). A summary of the fitted parameter posteriors is given below. In the present analysis, 200 posterior parameter sets were used along with the fitted parameters. To achieve reasonable statistics, we perform 1000 realisations for each parameter set in the stochastic model (the code and posterior parameters are attached).

Table 5: **Model parameterisation (posterior parameters).** Notation, a brief description, the median values, and the 95% certainty intervals of fitting parameters in Model W.

Notation	Description	Value		Unit
		Median	95% CI	
R_0	Basic reproduction number (NGM approach)	1.022	[1.009,1.065]	-
m_{eff}	Effective tsetse density			-
r	Relative bites taken on high-risk humans	3.883	[1.855, 8.257]	-
k_1	Proportion of low-risk people	0.9074	[0.7727,0.9761]	-
k_4	Proportion of high-risk people	$k_4 = 1 - k_1$		-
η_H	Treatment rate from stage 1 (1998 onwards)	4.290$\times 10^{-5}$	[2.564,6.698]$\times 10^{-5}$	days^{-1}
γ_{H0}	Treatment rate from stage 2 (pre-1998)	2.255 $\times 10^{-3}$	[1.669,3.145]$\times 10^{-3}$	days^{-1}
γ_H	Treatment rate from stage 2 (1998 onwards)	3.832$\times 10^{-3}$	[3.567,4.125]$\times 10^{-3}$	days^{-1}
Spec	Active screening diagnostic specificity	0.9996	[0.999,0.9999]	-
u	Proportion of passive cases reported	0.2444	[0.1478,0.3613]	-
d_{amp}	Relative improvement in passive stage 1 detection rate	0.8928	[0.7695,1.0195]	-
d_{steep}	Speed of improvement in passive stage 1 detection rate	1.103	[0.805,1.470]	-

2.5 Key updates of the model

This work provides a stochastic description of gHAT dynamics by considering random characteristics of human populations. It allows the population of human compartments to be integer variables and depicts the transitions between them with stochastic events. Whereas the original model presents a mean-field picture of smooth changes of human populations described by continuous variables (16; Crump et al.). Our current model accounts for the stochastic nature of different events such as human exposure, recovery, progression, and death. Therefore, individual simulations lead to different infection trajectories, whose averages follow the mean-field deterministic results. The discrete representation of human populations let us define elimination clearly, however in the ODE model an arbitrary threshold is set to define elimination. This stochastic model varies from that in (8) by including improvements to the stage 1 passive detection rate over time and by simulating health zone, rather than village-level population sizes.

References

[1] Artzrouni, M. and Gouteux, J.-P. (1996). A compartmental model of sleeping sickness in central Africa. *Journal of Biological Systems*, 4(04):459–477.

[2] Castaño, M. S., Ndeffo-Mbah, M. L., Rock, K. S., Palmer, C., Knock, E., Miaka, E. M., Ndung'u, J. M., Torr, S., Verlé, P., Spencer, S. E., Galvani, A. P., Bever, C., Keeling, M. J., and Chitnis, N. (2019). Assessing the impact of data aggregation in model predictions of hat transmission and control activities. *medRxiv*, page 19005991.

[3] Checchi, F., Chappuis, F., Karunakara, U., Priotto, G., and Chandramohan, D. (2011). Accuracy of five algorithms to diagnose gambiense human African trypanosomiasis. *PLoS neglected tropical diseases*, 5(7):e1233.

[4] Checchi, F., Filipe, J. A., Barrett, M. P., and Chandramohan, D. (2008). The natural progression of Gambiense sleeping sickness: what is the evidence? *PLoS neglected tropical diseases*, 2(12):e303.

[5] Checchi, F., Funk, S., Chandramohan, D., Haydon, D. T., and Chappuis, F. (2015). Updated estimate of the duration of the meningo-encephalitic stage in gambiense human African trypanosomiasis. *BMC research notes*, 8(1):292.

[6] Clausen, P., Adeyemi, I., Bauer, B., Breloeer, M., Salchow, F., and Staak, C. (1998). Host preferences of tsetse (Diptera: Glossinidae) based on bloodmeal identifications. *Medical and veterinary entomology*, 12(2):169–180.

[Crump et al.] Crump, R., Huang, C., Knock, E., Spencer, S., Miaka, E. M., Keeling, M. J., and Rock, K. S. Quantifying epidemiological drivers of gambiense human African Trypanosomiasis across the Democratic Republic of Congo. *In prep*.

[8] Davis, C. N., Rock, K. S., Miaka, E. M., and Keeling, M. J. (2019). Village-scale persistence and elimination of gambiense human African trypanosomiasis. *PLoS Neglected Tropical Diseases*, 13(10):1–15.

[9] Davis, S., Aksoy, S., and Galvani, A. (2011). A global sensitivity analysis for African sleeping sickness. *Parasitology*, 138(4):516–526.

[10] Gillespie, D. T. (1977). Exact stochastic simulation of coupled chemical reactions. *The journal of physical chemistry*, 81(25):2340–2361.

[11] Institut National de la Statistique, Ministère du Plan et Révolution de la modernité de la République Démocratique du Congo (2015). Annuaire statistique 2014. http://www.ins-rdc.org. [Online; accessed 22-January-2019].

[12] Mpanya, A., Hendrickx, D., Vuna, M., Kanyinda, A., Lumbala, C., Tshilombo, V., Mitashi, P., Luboya, O., Kande, V., Boelaert, M., et al. (2012). Should I get screened for sleeping sickness? A qualitative study in Kasai province, Democratic Republic of Congo. *PLoS neglected tropical diseases*, 6(1):e1467.

[13] OCHA Office for the Coordination of Humanitarian Affairs (Accessed May 2016). *Journées Nationales de Vaccination (JNV) Activités de vaccination supplémentaire , RDC*.

[14] Ravel, S., Grébaut, P., Cuisance, D., and Cuny, G. (2003). Monitoring the developmental status of *Trypanosoma brucei gambiense* in the tsetse fly by means of PCR analysis of anal and saliva drops. *Acta tropica*, 88(2):161–165.

[15] Rock, K. S., Ndeffo-Mbah, M. L., Castaño, S., Palmer, C., Pandey, A., Atkins, K. E., Ndung'u, J. M., Hollingsworth, T. D., Galvani, A., Bever, C., et al. (2018). Assessing strategies

against Gambiense sleeping sickness through mathematical modeling. *Clinical infectious diseases*, 66(suppl_4):S286–S292.

[16] Rock, K. S., Torr, S. J., Lumbala, C., and Keeling, M. J. (2015). Quantitative evaluation of the strategy to eliminate human African trypanosomiasis in the Democratic Republic of Congo. *Parasites & vectors*, 8(1):532.

[17] Rock, K. S., Torr, S. J., Lumbala, C., and Keeling, M. J. (2017). Predicting the impact of intervention strategies for sleeping sickness in two high-endemicity health zones of the Democratic Republic of Congo. *PLoS neglected tropical diseases*, 11(1):e0005162.

[18] Rogers, D. (1988). A general model for the African trypanosomiases. *Parasitology*, 97(1):193–212.

[19] Stone, C. M. and Chitnis, N. (2015). Implications of heterogeneous biting exposure and animal hosts on *Trypanosomiasis brucei gambiense* transmission and control. *PLoS computational biology*, 11(10):e1004514.

[20] The World Bank (Accessed 2015). *Data:Democratic Republic of Congo*.

[21] WHO (2013). Control and surveillance of human African trypanosomiasis. Technical Report 984.
